# Critical roles of plasma membrane dynamics and heterogeneity at the early stage of antigen-stimulated FcεRI signaling in mast cells

**DOI:** 10.1042/BST20253073

**Published:** 2025-10-09

**Authors:** Dishari Medda, Nirmalya Bag

**Affiliations:** Department of Chemistry, Indian Institute of Technology, Kharagpur, 721302, India

**Keywords:** FcεRI signaling, imaging fluorescence correlation spectroscopy, lipid rafts, membrane dynamics, super-resolution microscopy

## Abstract

Stimulated transmembrane (TM) signaling mediated by plasma membrane localized receptors is central to numerous cellular processes, and their dysregulation leads to pathological conditions. Antigen (Ag) stimulated clustering of high-affinity immunoglobulin E (IgE) receptor FcεRI and its functional coupling of selective signaling components such as kinases, but not phosphatases, in the early stage of mast cell signaling represents the general paradigm of TM signaling mediated by membrane receptors lacking kinase module. It has been long thought that plasma membrane organizational features, especially ordered regions and cortical cytoskeletons network, play crucial roles in efficient spatial sorting of the signaling components. In this review, we highlight the observations made by advanced imaging and spectroscopy techniques at high spatial and temporal resolution that essentially establish novel principles of plasma membrane ‘adaptivity’ in regulating the initial steps of stimulated mast cell signaling involving Ag cross-linked IgE-FcεRI receptor.

## Introduction

**Figure 1:**
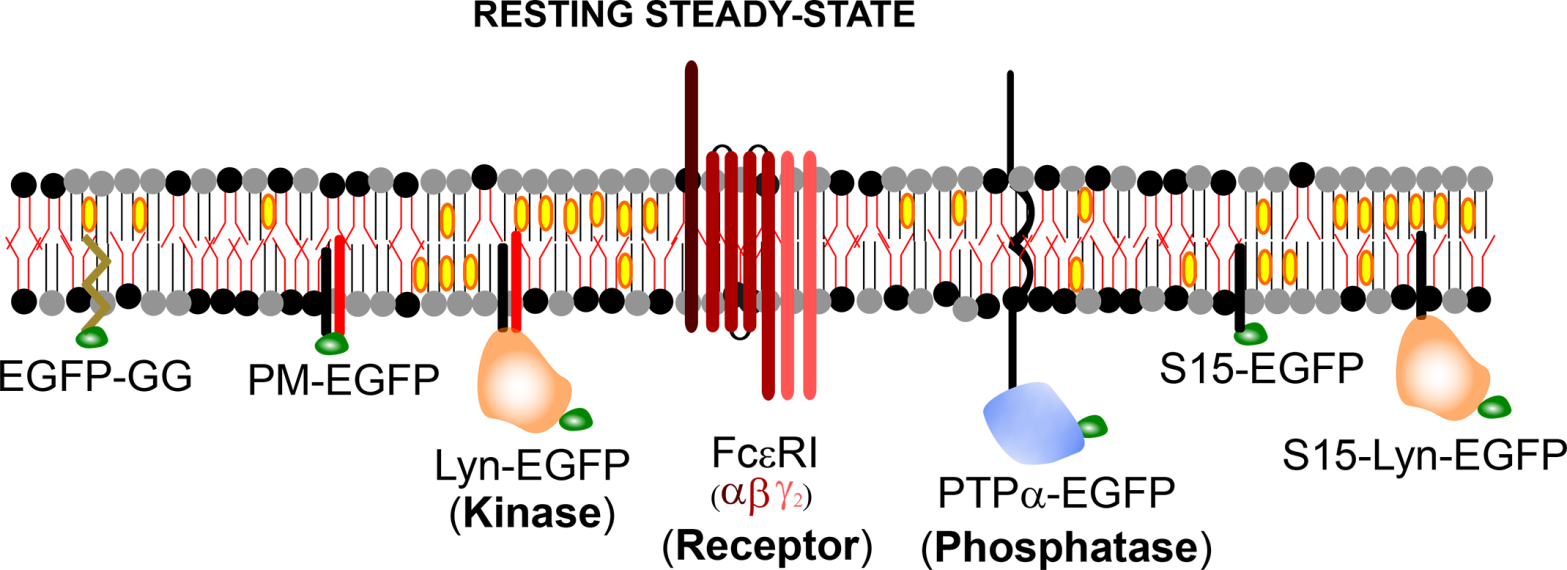
Membrane topology of various fluorescently labeled membrane components used to study the role of membrane heterogenity and dynamics on mast cell signaling. Here we showed EGFP as a fluorescent label (green; drawn not to scale). However, this may be replaced by other suitable fluorescent probes. Gray head group: saturated lipids, black headgroup: unsaturated lipids, yellow ellipse: cholesterol. EGFP, enhanced green fluorescent protein; GG, geranylgeranyl; PM, palmitoyl/myristoyl; PTPα, protein tyrosine phosphatase α.

High affinity receptor for immunoglobulin E (IgE), FcεRI, belongs to the multichain immune recognition receptor (MIRR) family [1]. Signal transduction elicited by extracellular triggering of MIRRs, notably T-cell and B-cell receptors (TCR and BCR), and FcεRI, share similar mechanisms [2]. Here, we focus on antigen (Ag) stimulated signaling of IgE bound FcεRI receptors in mast cells as occurs during allergy response [3]. The FcεRI receptor is composed of four co-associated α, β, and γ subunits in αβγ_2_ form, out of which the α chain has extracellular ligand binding module and the signaling chains, namely β and γ chains, have cytosolic immunoreceptor tyrosine-based activation motifs (ITAMs) (Figure 1) [1,4,5]. Sensitization of FcεRI by high affinity binding of IgE at the extracellular region to its α subunit, followed by cross-linking of IgE-FcεRI complex by soluble, multivalent Ag, generates receptor nanoclusters [3]. These cross-linked receptor nanoclusters are then activated by tyrosine phosphorylation at the cytosolic ITAMs of both β and γ subunits by Lyn, a src family non-receptor tyrosine kinase localized at the cytosolic (inner) leaflet of the plasma membrane [4]. The phosphorylated receptors then recruit soluble Syk kinase at the inner leaflet to initiate the subsequent highly orchestrated downstream processes eventually leading to functional responses such as degranulation [3].

The signaling components (both plasma membrane localized and intracellular) involved in stimulated mast cell signaling cascade are mostly well established [3]. The plasma membrane-localized components involved at the early stage of signaling include the receptor itself, a lipid-anchored kinase (Lyn), and a transmembrane (TM) tyrosine phosphatase. However, the principles governing the functional coupling of Lyn and cross-linked receptors (as happens after stimulation), but not of Lyn and uncross-linked receptors (in the resting state), even though all signaling components (receptor, kinase, and phosphatase; Figure 1) are simultaneously present at the plasma membrane, are being investigated to date. There are several models, reviewed elsewhere [6,7], proposed to explain how Lyn initiates receptor phosphorylation outperforming dephosphorylation by phosphatases. These models primarily differ in the spatial positioning of the signaling components in resting conditions and their dynamic reorganization after stimulation. The physico-chemical requirements of these spatio-temporal changes of the signaling components essentially dictate whether protein-based or lipid-based interactions drive functional kinase/receptor coupling according to a given model.

An attractive lipid-centric model, emerged in the late 1990s [8], invokes a pivotal role of ordered membrane regions [9–11], colloquially known as membrane rafts [12], in the initial coupling of Lyn and cross-linked receptors required for mast cell activation [13–15]. These ordered regions are likely to be nanoscopic in the live cells since they are not detectable by optical microscopy [16–19] while complementary spectroscopic signatures confirm their presence [20–24]. Their biophysical properties, as examined by spectroscopic methods, resemble those of the liquid ordered (Lo) phase of model lipid bilayers exhibiting liquid–liquid phase separation into coexisting Lo and liquid disordered (Ld) phases [25–27]. In live cells, these Lo-like regions are enriched with saturated lipids and are spatially segregated from the surrounding Ld-like regions which are enriched with unsaturated lipids [12]. Further, cholesterol and cortical cytoskeleton are reported to control the Lo/Ld phase-like organization in plasma membranes [28–31]. This structural phase-like organization of the plasma membrane provides means for the functional membrane components to spatially and dynamically sort themselves based on their partitioning preference for optimal efficiency [32,33]. For example, Lyn is anchored to the plasma membrane via saturated palmitoyl/myristoyl (PM) acyl chains and thus preferentially partitions into the Lo-like regions [34–37]. By contrast, TM phosphatase, for example protein tyrosine phosphatase α (PTPα), experiences unfavorable interactions in the relatively tightly packed Lo-like regions and hence prefers Ld-like regions [36,38]. The intrinsic preference of the signaling components is the key attribute of the lipid-centric model of functional kinase/receptor coupling. According to this model, in the resting steady state, phosphorylation of uncross-linked IgE-FcεRI receptors by Lyn kinase is, on average, counter-balanced by their dephosphorylation by TM phosphatase; and thereby the level of the receptor phosphorylation is maintained below the functional threshold. Upon stimulation, the Ag cross-linked receptors are surrounded by relatively stable Lo-like environment into which Lyn kinase, but not TM phosphatase, preferentially partitions. This spatial sorting allows Lyn kinase to interact with the cross-linked receptors more frequently than that of phosphatases shifting receptor phosphorylation/dephosphorylation balance towards more phosphorylation [38,39]. This eventually leads to the level of receptor phosphorylation above the functional threshold, which is a prerequisite for downstream signal transduction. Here, we review the current status and potential extension of this model based on the evidence provided by two state-of-the-art techniques, namely two-color Single Molecule Localization Microscopy (SMLM), sometimes called fluorescence localization microscopy, and multiplexed Imaging Fluorescence Correlation Spectroscopy (ImFCS) (Table 1), after briefly describing the early investigations in this topic.

**Table 1:** Comparison between pc-SMLM and ImFCS methods

Parameters	pc-SMLM	ImFCS
Mode of measurement	Spatial localization of membrane probes using spatial correlation analysis	Macroscopic diffusion of membrane probes using temporal correlation analysis
Spatial resolution	~20 nm	~250 nm
Temporal resolution	1 sec	1 ms
Fluorophores	A pair of fluorescent probes having photophysical properties suitable for single molecule localization microscopy	Any standard fluorophore used in conventional optical microscopy
Instrumental set up	TIRFM with sensitive camera	TIRFM with sensitive and high-speed camera
Cautions during data analysis	Requires elimination of overcounting to avoid errors	Requires photobleach correction to avoid errors
Major advantage	Cross-correlation amplitude mirrors degree of colocalization between two membrane probes directly.	Unprecedented data statistics determine very modest differences (5%) in probe diffusion at various membrane states, which are generally difficult to resolve by existing methods.

TIRFM, Total Internal Reflection Fluorescence Microscopy .

### Initial evidence on the role of Lo-like environment in stimulated mast cell signaling

The quest to understand the importance of lipid-based interactions in mast cell signaling was started around the time when the concepts of detergent-resistant membrane (DRM) fraction and its enrichment with glycosylphosphatidylinositol (GPI)-anchored proteins and order-promoting lipids started to become experimentally tested [9,10,40]. The Baird group presented a series of articles with evidence that: (a) Ag cross-linked FcεRI receptors are detergent-resistant and the association of the receptor to DRM is independent of ITAM phosphorylation [14], (b) Lyn kinase in stimulated cells is isolated from DRM [13] and the saturated PM lipid anchor of the kinase drives its partitioning into these ordered membrane regions [35,41], (c) specific kinase activity of Lyn isolated from DRM is about five times larger than those isolated from detergent-soluble membrane fraction [39], and (d) decrease in stimulated phosphorylation of Ag cross-linked FcεRI receptors when a PM-anchored phosphatase, that associates with DRM, is co-expressed with Lyn kinase compared with when a detergent-soluble TM phosphatase is co-expressed in a reconstituted system [38]. These biochemical studies conclude that signaling of cross-linked FcεRI receptors occurs from DRM in which the phosphorylating Lyn kinase, but not the TM phosphatase, is present [14,35,38,39,42].

Sheets and co-workers employed fluorescence lifetime imaging microscopy and fluorescence anisotropy of lipid probes directly on mast cell plasma membranes after extensive micron-scale patching of FcεRI receptors by polyclonal antibodies [43,44]. Their observations allude to the ordered nature of membrane regions proximal to the cross-linked receptor patches. The stability of these ordered regions was also shown to be dependent on cholesterol [42] and cortical cytoskeleton [44]. Overall, ordered regions of plasma membrane and lipid-driven partitioning preference of plasma membrane-localized key signaling components (i.e. Lyn kinase and TM phosphatase) play crucial roles at the initial stage of stimulated TM signaling in mast cells. Notably, Lo-like or ordered regions were also implicated in stimulated signaling through immunoreceptors, including TCR [45–47], BCR [37,48,49], and toll-like receptor (TLR) [50].

It should be noted that non-physiological sample preparation conditions (e.g. harsh detergent treatments used for DRM isolation, patching of receptors by extensive cross-linking etc.), technical challenges (e.g. optically unresolved spatial dimension), and indirect nature of these measurements (e.g. cholesterol extraction) attracted criticisms on the plausibility of the importance of lipid-driven mechanisms in this context [51,52]. In fact, the presence of ordered regions as plasma membrane organizational features was doubted because of these reasons. As discussed below, recent advances in live cell compatible imaging and spectro-microscopic techniques, along with less perturbing conditions, were able to address most of these issues and provided better insights into various interaction modalities among the plasma membrane localized mast cell signaling components.

### Pair correlation SMLM (pc-SMLM) provides direct evidence of lipid-driven nanoscopic co-localization of signaling components

Veatch and co-workers led the applications of SMLM to illuminate nanoscale organization of signaling components in stimulated steady states of immune cells [53]. The principles of SMLM are the same as single-molecule localization microscopy techniques such as photoactivatable localization microscopy (PALM) or stochastic optical reconstruction microscopy (STORM) [54–56], but here it was applied to localize nanoclusters containing multiple molecules. The super-resolved images (spatial resolution ~20 nm) obtained from single-color SMLM measurements can be spatially correlated to determine the average size of the fluorescent species. Ag cross-linking of fluorescently labeled FcεRI receptors yields nanoclusters having an average size around 70 nm at the stimulated steady state in rat basophilic leukemia (RBL-2H3) mast cells [57]. Both stimulated receptor nanoclustering and subsequent Ca^2+^ mobilization were further shown to be dependent on cholesterol content.

**Figure 2:**
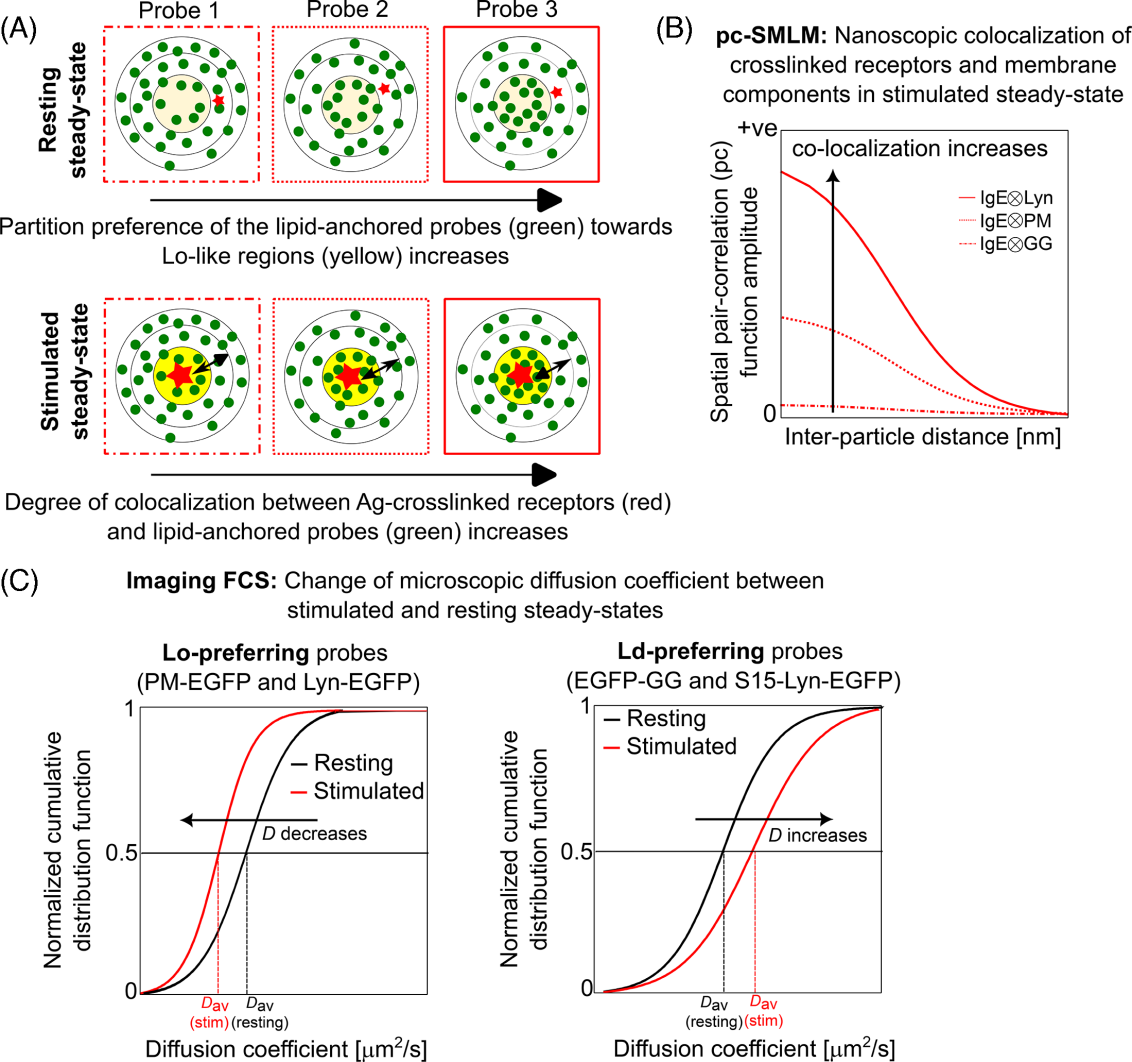
Principles of pair-correlation Single Molecule Localization Microscopy (pc-SMLM) and Imaging Fluorescence Correlation Spectroscopy (ImFCS) in relation to the detection of lateral organization of fluorescently labeled membrane components. (**A**) A schematic snapshot of the spatial distribution of the membrane components (lipid-anchored probes: green circle, receptor: red asterisk) as per their inherent dynamic partitioning behavior. The Lo-preference of the probes increases as: probe 1 > probe 2 > probe 3. For example, Lo-preference of fluorescently labeled lipid-anchored probes is ranked as: Lyn ~ PM [K_p_=(Lo)/(Ld)=1.5] > S15 (K_p_=0.44) ~ S15-Lyn > GG (K_p_=0.29) [37]. In resting steady state, Lo/Ld-like membrane organization is less distinctive (light yellow: Lo-like region and white: Ld-like region). In stimulated steady state, this phase-like organization is more distinctive (dark yellow: Lo-like region and white: Ld-like region). The Lo-like regions locally surround the cross-linked receptor nanocluster (red) and are locally surrounded by Lo-like regions into which the membrane components dynamically partition as per their inherent Lo-preference (top panel). (**B**) Larger amplitude of spatial pair-correlation function between the cross-linked receptor and a given membrane component measured by pc-SMLM reflects stronger nanoscale colocalization. (**C**) The changes in the stability of phase-like organization between resting and stimulated steady states affect the diffusion behavior of the membrane probe measured by ImFCS. The diffusion of an Lo-preferring probe (probe 1 in Figure 2A) decreases, while that of an Ld-preferring probe (probe 3 in Figure 2A ) increases in stimulated conditions.

In a follow-up study [58], the authors performed two-color SMLM to evaluate nanoscale co-localization between cross-linked receptors and other protein and lipid probes. They implemented the principles of image cross-correlation spectroscopy (ICCS) [59], which quantifies the probability of finding two probes as a function of their spatial separation, on two-color SMLM images to generate spatial cross-correlation function, which is also called pair-correlation function of SMLM (pc-SMLM) [60,61]. If two membrane components are randomly distributed with respect to each other, the pc-SMLM amplitude is zero. In contrast, the pc-SMLM amplitude is positive if the components are co-localized at nanoscopic length scale. The amplitude of pc-SMLM increases with the degree of co-localization (Figure 2A–B). In the current context, the authors performed pc-SMLM analyses [58] between fluorescently labeled Ag cross-linked IgE-FcεRI receptor and different membrane probes, including fluorescently labeled Lyn, saturated PM lipid [35], and unsaturated geranylgeranyl (GG) lipid [35] probes as schematically shown in Figure 2A. The pc-SMLM amplitude was positive for the cross-correlation of cross-linked receptor and either of Lyn and PM probes (both order preferring), but not for the GG probe (disorder preferring) (Figure 2B). Since PM is the lipid anchor of Lyn (Figure 1) and is non-functional, its co-localization with cross-linked receptors cannot be driven by protein-based interactions. Rather, its association with the ordered regions surrounding cross-linked receptors, and hence positive pc-SMLM amplitude, is stemmed from its inherent lipid-driven partitioning preference [37,41]. The partitioning preference of the unsaturated lipid probe, GG, into the disordered regions [37,41] does not render any co-localization with the cross-linked receptors as expected [37]. These nanoscopic investigations unambiguously establish the presence of ordered regions around cross-linked receptor nanoclusters in stimulated cells.

The same study [58] also showed that pharmacological inhibition of actin polymerization influences the pc-SMLM amplitude of cross-linked receptor and PM probe, indicating a regulatory role of cortical cytoskeleton on the stability of the ordered regions in stimulated RBL-2H3 cells. The authors compared the colocalization of cross-linked receptors with lipid probes in cytochalasin D (inhibits actin polymerization by capping the barbed end) or latrunculin A (induces actin depolymerization) treated cells with untreated cells. They observed that perturbation of actin polymerization enhances colocalization of cross-linked receptors and order-preferring membrane components. In another study, Bag et al. showed that cytochalasin D treatment stabilizes phase-like organization in resting RBL-2H3 cells [62]. Taken together, stabilization of phase-like organization in resting cells (before receptor cross-linking), resulting from the inhibition of actin dynamics, promotes colocalization of cross-linked receptor and order-preferring components in stimulated cells. Note that actin-dependent stabilization of phase-like organization may be cell-line dependent. These results in RBL-2H3 cells are also consistent with previous functional studies [63,64].

Principles of spatial autocorrelation and pair-correlation analyses in scanning electron microscopy (SEM) data were also used to evaluate spatial organization of Ag cross-linked IgE-FcεRI and Lyn kinase [65]. However, the relatively large size and non-specific interactions of gold-conjugated secondary antibodies used for receptor labeling in SEM measurements are potential sources of error in the size estimation of small nanoclusters. These labeling artifacts were largely avoided in SMLM studies by using fluorescent protein labels. However, pc-SMLM was restricted to the measurements on stimulated cells, while complete understanding of the changes in the membrane organization of the signaling components and its functional relevance requires knowledge of both resting and stimulated steady states. Furthermore, additional care should also be taken to avoid overcounting problems in SMLM image analysis [66].

### Imaging FCS (ImFCS) reveals modular lipid-based, protein-based, and steric interactions among signaling components

One of us (N.B.) while working in the Baird-Holowka laboratory (Cornell University, NY) applied ImFCS, which analyzes temporal autocorrelation of fluorescence fluctuation at each pixel [67] (length scale: 160–320 nm depending on the instrumental setup) of the plasma membrane to compare diffusion properties of a dozen of membrane probes in resting and stimulated steady states to illuminate various modes of interactions in the context of mast cell signaling [36]. Generally, an ImFCS measurement on a given cell yields several hundred diffusion coefficient (*D*) values [68], and pooling data from tens of cells would provide more than 10,000 *D* values in a given condition. The average of these *D* value (*D_av_*) is very precise (the standard error of the mean is less than 1%). Evaluation of statistical difference between two data sets having more than 10,000 data points per set requires additional caution since the *P*-value decreases exponentially with the number of data points [69]. This might lead to false interpretation of statistical difference for very large sample size. As an alternative, we chose random sub-samples by bootstrapping from each data set using 50% of all data and compared them statistically. We repeated this strategy more than 30 times between two given data sets to determine whether the original data sets are statistically different. Using this approach, we were able to comprehensively detect small changes of *D_av_* values (even 5%) of membrane probes between resting and stimulated steady states [36,62]. We then measured diffusion of a range of judiciously chosen mutant probes in both resting and stimulated steady states to establish this approach and later connected their interpretations from the diffusion analysis to the functional results [36]. We used this statistical robustness of diffusion data obtained from ImFCS to delineate organization of the plasma membrane sensed by a diffusing membrane probe. As illustrated in Figure 2A, if the ordered regions of the plasma membrane become more stable locally after receptor stimulation, an order-preferring lipid probe will show slower diffusion since it will spend relatively more time in the ordered (more viscous) regions. A disorder-preferring probe, by contrast, will show faster diffusion under the same circumstances since it will be spending more time in the disordered (less viscous) regions. For reference, the *D_av_* of membrane probes in Lo and Ld phases in phase-separated giant unilamellar vesicles is 0.2–0.9 μm^2^/s and 4–6 μm^2^/s, respectively [70]. The difference in the *D_av_* value between Lo-like and Ld-like phases in cell-derived giant plasma membrane vesicles is somewhat smaller (1.8 and 5.6 μm^2^/s respectively) [21]. Simply stated, if the ordered and disordered regions of the plasma membrane become more distinctive in stimulated steady state, there will be a net decrease and increase in *D_av_* of order-preferring and disorder-preferring probes, respectively (Figure 2C).

It is worth pointing out that the Lo/Ld-like organization in the resting state is transient (in the milliseconds range) [20,21,23,71]. Since the timescale of membrane diffusion of lipid probes is also in the millisecond range, the ordered and disordered regions appear to be less distinctive for a diffusing probe. When Lo/Ld-like organization is stabilized (i.e. much longer than milliseconds) by some external perturbation, the diffusion trend of Lo- and Ld-preferring probes goes in opposite directions even in the resting state. For example, inhibition of actin polymerization results in a decrease in *D_av_* (measured by ImFCS) of fluorescently labeled PM probe, while that of GG probe increases in resting cells [62]. This supports the existence of Lo/Ld-like regions in the resting cells, and their size and dynamics are regulated by cortical cytoskeleton, as also demonstrated in model systems [72–74] and live cells [75] previously.

As reported here [36], compared with the resting steady state, the *D_av_* of enhanced green fluorescent protein (EGFP)-labeled PM and Lyn probes (both Lo-preferring) decreases by 8–10% in the stimulated steady state, while the opposite trend was observed for EGFP-labeled GG and S15 probe (both Ld-preferring) (Figure 2C). The lipid probes, PM-EGFP, EGFP-GG, and S15-EGFP (Figure 1), only undergo lipid-based interactions since they do not possess any protein-interacting modules. Therefore, the observed opposite diffusion trends of Lo- and Ld-preferring probes are driven by their respective lipid-based partitioning preferences. Akin to the pc-SMLM measurements described above [58], the behavior of the lipid probes establishes that the ordered and disordered regions are more distinctive in stimulated steady state [62].

PM-EGFP and Lyn-EGFP have the same lipid anchors, while the latter has multiple protein-interacting modules (SH2, SH3, and kinase modules). Both pc-SMLM and ImFCS studies showed that ordered regions are locally concentrated around the cross-linked receptor (discussed above). Therefore, we asked whether lipid-based interactions are required for the partitioning of the Lyn kinase into these receptor-proximal ordered regions to facilitate functional interactions. For this, we created a chimeric Lyn probe, namely S15-Lyn-EGFP, which is Ld-preferring but possesses all protein-interacting modules of Lyn kinase [36] (Figure 1). Interestingly, the diffusion of S15-Lyn-EGFP becomes faster after receptor stimulation, resembling the behavior of Ld-preferring lipid probes such as S15-EGFP and EGFP-GG. In a reconstituted system containing minimally required plasma membrane signaling components (receptor, kinase, and phosphatase in Chinese Hamster Ovary cells), Lyn-EGFP, but not S15-Lyn-EGFP, phosphorylates cross-linked receptor confirming functional importance of Lyn’s Lo-preference. Put together, lipid-based interactions of Lyn-EGFP are essential for its biophysical and functional coupling with Ag cross-linked FcεRI receptors and their consecutive phosphorylation.

Lyn has three protein-interacting modules having the following general functions: (i) SH2 module binds to phosphotyrosine (pTyr), (ii) SH3 module binds to polyproline motifs, and (iii) kinase module participates in phosphorylation reactions. It was previously shown that pharmacological inhibition of Lyn kinase activity by PP1 in the resting state decreases the amounts of stimulated receptor clustering but does not completely eliminate its co-localization with Lyn [65]. We therefore tested whether the interacting modules contribute to the coupling of Lyn kinase and stimulated receptors by creating EGFP-tagged Lyn probes having inhibitory point mutation in each of the modules [36,76] separately. These mutant probes are Lo-preferring since their plasma membrane anchor (saturated PM chains) is the same as the Lyn-EGFP. If a module does not participate in the additional interactions in stimulated cells, the corresponding probe would not show a stimulated decrease in its *D_av_*. We found that *D_av_* of all mutants decreases in stimulated cells, confirming their interactions with the cross-linked receptors [36]. Notably, the *D_av_* of Ld-preferring mutant Lyn probe, S15-Lyn-EGFP, increases in the stimulated cells. This means Lo-preferences of Lyn and its mutants are essential for the interactions with Ag cross-linked receptors, which result in a decrease in their respective *D_av_*. A careful examination of the net change of *D_av_* of various probes also reveals that none of the probes having point mutation in the protein-interacting modules has the same degree of *D_av_* change as the Lyn-EGFP probe [36]. Therefore, protein-based interactions via SH2, SH3, and kinase modules are involved in the functional coupling of Lyn and cross-linked receptors [36]. The same conclusion was reached when the degree of recruitment of these mutants was compared with the Lyn-EGFP on the micron-scale Ag-patterned substrates used for receptor cross-linking [76].

We further observed [36] that the exclusion of the TM phosphatases from the proximity of the cross-linked receptors was driven by two factors. Firstly, TM phosphatases preferentially partition into the disordered (less viscous) and less tightly packed regions of the plasma membrane. Secondly, the immobile Ag cross-linked receptor nanoclusters in stimulated steady state act as a steric obstacle for the diffusion of various TM probes. Therefore, the Ag cross-linked receptors are spatially protected from TM phosphatases by their opposite preferences between ordered and disordered regions as well as by steric factors diminishing the probablity of receptor dephosphorylation.

**Figure 3:**
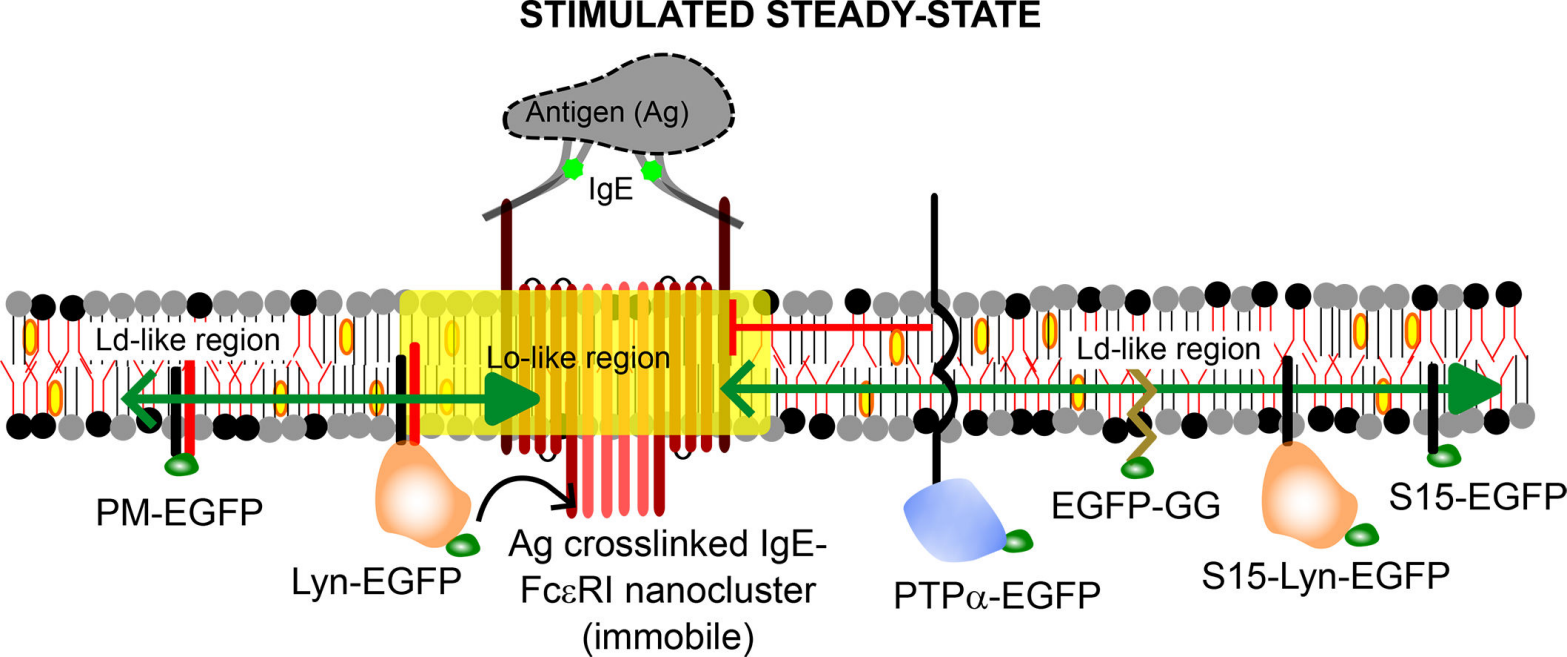
Minimal biophysical model to describe spatial organization of membrane components in stimulated steady state necessary for functional Ag-stimulated FcεRI signaling. The stabilized Lo-like region (yellow) around the Ag cross-linked IgE-FcεRI receptors and Ld-like regions away from them. The dynamic partition preference of the membrane probes is shown by a green double-headed arrow: thick head is pointed towards the preference [PM, Lyn: Lo preference; K_p_>1 (Figure 2A); PTPα, GG, S15, S15-Lyn: Ld preference; K_p_<1 (Figure 2A)]. Steric hindrance of the mobile TM components (e.g. PTPα) by the immobilized receptors is shown by red left-blocking solid line. The arrow indicates net phosphorylation of the IgE-FcεRI receptor cluster by Lyn kinase facilitated by the Lo preference of the latter. Here we showed EGFP as fluorescent label (green). Note that all probes, except for the clustered receptors, remain mostly mobile in stimulated steady state.

Overall, lipid-based, protein-based, and steric interactions between receptor, kinase, and phosphatases occur in the stimulated steady states (Figure 3). These interactions centrally leverage the biophysical properties of the plasma membrane, including its capacity for phase-like separation into ordered and disordered regions and diffusion of TM probes present in a fluid two-dimensional (2D) system containing immobilized obstacles. The synergy of these interactions defines a membrane state to orchestrate an optimal degree of signaling reactions.

### Importance of resting state plasma membrane order in stimulated mast cell signaling

Yang et al. tested whether altering resting state order by adding order-supporting or disorder-supporting lipids [77–79] in the plasma membrane changes stimulated functional responses in the mast cells [64]. They observed a strong positive correlation between stimulated recruitment of Syk kinase to the plasma membrane (a membrane proximal functional consequence of Ag cross-linked FcεRI clustering) to resting state order. However, Syk recruitment was not observed in the absence of receptor cross-linking even though the membrane order was increased. This means that increasing membrane order (before stimulation) does not induce aberrant Ag-independent signaling responses in mast cells. However, more ordered resting membrane likely facilitates in the modulation of the stimulated distinctiveness between Lo- and Ld-like regions. These results further support an intricate association of membrane order in stimulated FcεRI signaling. These results are in the same line with the observed increase in stimulated nanoscale co-localization between Ag cross-linked IgE-FcεRI/PM and stimulated phosphorylation of FcεRI receptors in cells pre-treated (i.e. in resting condition) with inhibitors of actin polymerization [58]. As noted earlier, inhibition of actin polymerization stabilizes the Lo/Ld-like organization in the inner leaflet in the resting cells [62]. Simulated responses are subsequently enhanced as a result of this increased ordering in the resting state. Overall, the resting plasma membrane organization of signaling components is poised to respond to extracellular Ags.

## Conclusion

The present minireview focuses on the dynamic membrane reorganization that takes place when mast cells get activated by stimulation of the IgE-FcεRI complex by soluble, multivalent Ag. The balance of phosphorylation and dephosphorylation of the receptor in resting and stimulated states plays pivotal roles in this process [38]. We discussed here the most recent results from high-resolution microscopy and spectroscopy methods, namely pc-SMLM and ImFCS, to develop the current picture on the regulatory roles played by resting-state membrane order as well as other membrane organizational features, including lipid-driven Lo/Ld-like organization and immobile solid-like diffusion obstacles at the early stage of Ag-stimulated FcεRI signaling (Figure 3).

While the earlier models [6,7] emphasize specifically either on lipid-based or on protein-based interactions, our most recent observations by high-resolution spectroscopy and microscopy collectively present an integrated biophysical model (Figure 3) where physical properties of the plasma membrane play decisive roles in facilitating signaling-competent protein-based interactions. Lyn kinase undergoes enhanced encounters with the cross-linked receptors by its inherent preference to dynamically partition into the Lo-like regions along these receptors. By contrast, the Ld-preferring TM phosphatase is excluded from these regions. In addition, the immobilized cross-linked receptors themselves act as steric obstacles for any TM probe, including the phosphatase. These two factors collectively reduce phosphatase’s interactions with the cross-linked receptors. Under these circumstances, the kinase-mediated receptor phosphorylation is not balanced by phosphatase-mediated dephosphorylation, leading to net phosphorylation above non-functional threshold. This mechanism does not occur in the resting steady state due to the absence of optimally stable Lo/Ld-like organization and immobile receptor nanoclusters. Overall, the tunability of the plasma membrane to adapt a slightly more distinctive Lo/Ld-like organization in stimulated conditions, directly or indirectly triggered by the formation of immobile receptor nanoclusters, is therefore the central element to this model. The subtlety of this ‘on-demand’ reorganization of the plasma membrane made it difficult to detect experimentally previously, which downplayed its significance in TM signaling. It will be interesting to interrogate the chemical and mechanical driving forces behind the formation and sustainability of the local ordered regions surrounding Ag cross-linked receptors in the future. Overall, we anticipate that the holistic approach evaluating diffusion changes between resting and stimulated states (by ImFCS) and nanoscale colocalization of signaling components (by pc-SMLM) (Figure 2) as discussed in this review will be widely employed in discovering novel concepts of functional membrane organization in cellular signaling.

**Perspectives**

- Signal transduction is critically regulated by two-dimensional liquid ordered/liquid disordered (Lo/Ld) phase-like separation of the plasma membrane. Since the FcεRI signaling pathway described here is similar to those of more clinically relevant T-cell and B-cell receptor systems, it is likely that plasma membrane phase-like separation is generally important in immunity.
- At the early stage of mast cell signaling, the plasma membrane adapts slightly more stable Lo-like regions after antigen-stimulated receptor nanoclustering and immobilization. Dynamic co-condensation of selective signaling components (stimulated receptor and kinase) within these ordered platforms is essential for transmembrane and downstream signaling.
- The generality of the regulatory role played by phase-like organization in cellular signaling mediated by plasma membrane localized receptors, especially those implicated in alarming diseases, will continue to be investigated in the future using cutting-edge techniques such as imaging fluorescence correlation spectroscopy and pair correlation SMLM. Detailed knowledge of these lipid-driven mechanistic principles would then likely translate into next-generation therapeutic interventions.

## Open Access

This article has been published open access under our Subscribe to Open programme, made possible through the support of our subscribing institutions, learn more here: https://portlandpress.com/pages/open_access_options_and_prices#conditional

## Abbreviations

Ag: antigen
BCR: B-cell receptor
DRM: detergent-resistant membrane
EGFP: enhanced green fluorescent protein
GG: geranylgeranyl
ICCS: image cross-correlation spectroscopy
ITAMs: immunoreceptor tyrosine-based activation motifs
IgE: immunoglobulin E
ImFCS: imaging fluorescence correlation spectroscopy
Ld: liquid disordered
Lo: liquid ordered
MIRR: multichain immune recognition receptor
PALM: photoactivatable localization microscopy
PM: palmitoyl/myristoyl
PTPα: protein tyrosine phosphatase α
SEM: scanning electron microscopy
SMLM: single molecule localization microscopy
STORM: stochastic optical reconstruction microscopy
TCR: T-cell receptor
TLR: Toll-like receptor
TM: transmembrane
pc-SMLM: pair correlation SMLM
